# A Comprehensive Review on Chemical Structures and Bioactivities of *Ostropomycetidae* Lichens

**DOI:** 10.3390/jof11050369

**Published:** 2025-05-09

**Authors:** Yunhui Wang, Chengyue Hao, Shuhao Jiang, Yanhu Ju, Wei Li, Zefeng Jia

**Affiliations:** College of Agriculture and Biology, Liaocheng University, Liaocheng 252059, China; 15314150512@163.com (Y.W.); sdhcy15169956173@163.com (C.H.); jsh20000522@163.com (S.J.); juyanhu@lcu.edu.cn (Y.J.)

**Keywords:** lichenized fungi, Lecanoromycetes, Ascomycota, secondary metabolites, bioactivities

## Abstract

Lichenized fungi, recognized as an ecologically vital and pharmaceutically promising resource, hold substantial value in both environmental conservation and medicinal applications. As the second largest subclass within the lichen-forming fungi of Lecanoromycetes, *Ostropomycetidae* emerged as a critical reservoir of bioactive secondary metabolites. Current research has revealed that these secondary metabolites demonstrate remarkable bioactivities, positioning them as potential sources for novel pharmaceutical compounds. Despite considerable progress in characterizing chemical constituents and evaluating bioactivities within this subclass, a systematic summary of these discoveries remains absent. This review synthesizes the lichenochemical research progress, providing critical evaluations of 202 structurally characterized compounds from *Ostropomycetidae* lichen species over recent decades. These *Ostropomycetidae*-derived compounds cover the phenols, polyketides, fatty acids, terpenoids, steroids, and non-ribosomal peptides, and exhibit diverse bioactivities including antitumor, anti-inflammatory, antibacterial, antifungal, antiviral, antioxidant, anti-angiogenic, anti-neurodegenerative diseases, antitubercular, anti-herbivore, and antitrypanosomal, and so on. The aim of this review is to establish a robust chemodiversity framework and to offer strategic guidance for targeted exploration of lichen-derived drug candidates in the biological resources of *Ostropomycetidae* lichens.

## 1. Introduction

Lichenized fungi (lichens) have been known for a long time as one type of excellent symbiotic association. More than 19,000 species of lichenized fungi have been reported, making up 17% of the known 110,000 fungal species and 27% of the known Ascomycota. Lichens grow practically on and within rocks, on soil and tree bark, and on almost any inanimate object, and so on. In nature, lichens grow very slowly [1,2]. The unique composition of lichens, containing fungi and algae or cyanobacteria, bestows them with both environmental sensitivity as bioindicators and remarkable stress resistance through physiological adaptation and chemical diversification [3]. The taxon-specific distribution patterns of chemical substances have also become fundamental to lichen taxonomy and systematics [4].

More than 800 unique secondary metabolites are currently known in lichens, with the number of compounds obtained under axenic culture conditions being significantly lower. Major compound types include depsides, depsidones, depsones, dibenzofurans, usnic acid derivatives, terpenoids, xanthones, chromones, and quinones, with structural diversity observed across most lichen species [2]. The photobiont symbiont, commonly comprising *Trebouxia* and *Trentepohlia* (chlorophyta) or *Nostoc* (cyanobacteria) along with associated microbiota, demonstrates the tremendous biosynthetic ability and potential to produce a range of potentially secondary metabolites [5,6,7,8,9]. Recent pharmacological investigations have revealed multifaceted bioactivities in lichen-derived compounds, including antimicrobial, antioxidant, antiviral, and anticancer properties. Notably, several metabolites exhibit selective cytotoxicity against malignant cells, either as standalone agents or chemotherapeutic adjuvants [10]. Secondary metabolites from lichens are worthy of further investigation in terms of their potential therapeutic applicability, including a better understanding of their mechanisms of action, which positions them as promising drug candidates [11]. These research advancements suggest that the lichens can be productively used in pharmaceutical relevance because of their possible activities reported [2]. These chemicals make up a treasure trove of pharmacologically active ingredients that are important for human life and health.

**Figure 1:**
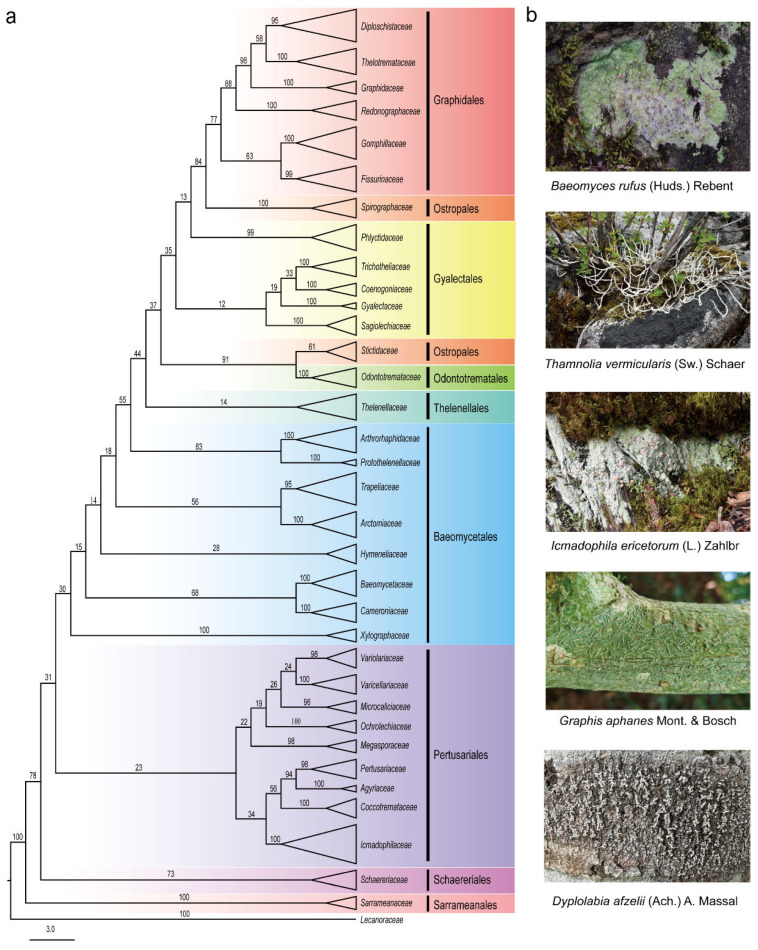
Phylogenetic analysis and the morphology of lichen in the *Ostropomycetidae* subclass. (**a**) Phylogenetic tree analysis of taxa at the family level. (**b**) Morphological characteristics of the lichen thallus of some species in *Ostropomycetidae*.

**Figure 2:**
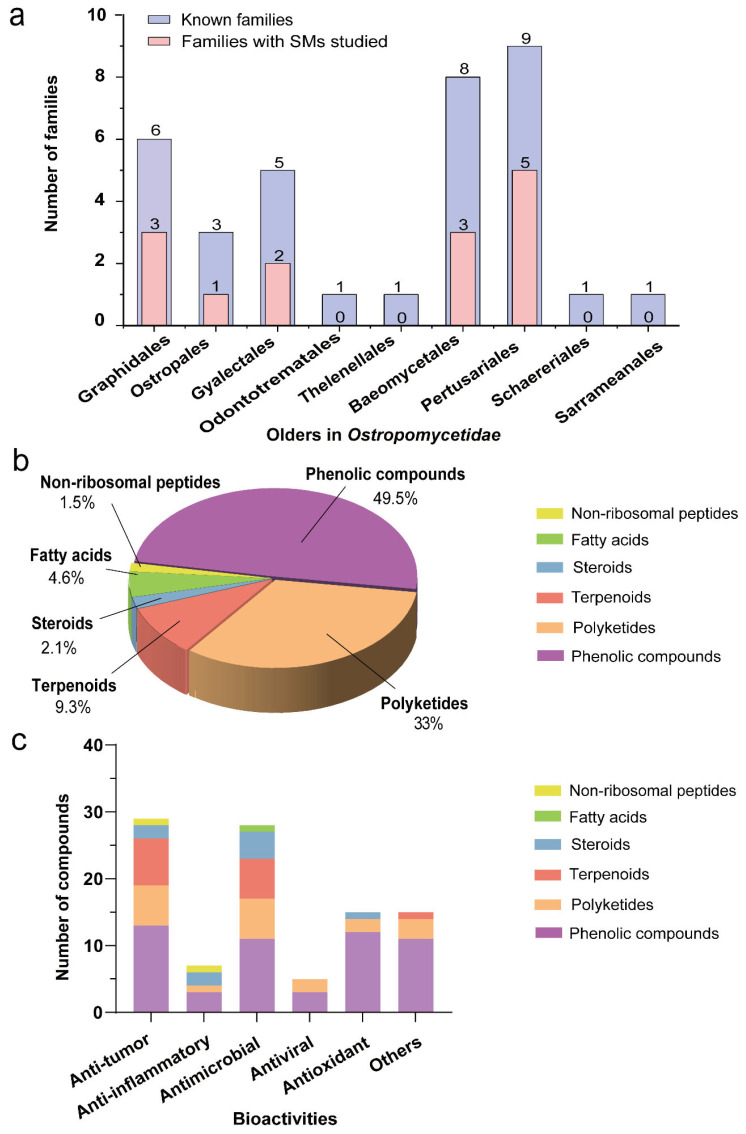
Bird’s-eye view of compounds from *Ostropomycetidae*. (**a**) The number of families studied to produce compounds in *Ostropomycetidae*. (**b**) The number of compounds of different structural types. (**c**) Different types of compounds with bioactivities.

*Ostropomycetidae* constitutes the second largest subclass within Lecanoromycetes [12]. It includes lineages with mostly non-amyloid asci but often amyloid hamathecium or ascospores. The predominant type of ascoma ontogeny in the subclass is hemiangiocarpous. All the Lecanoromycetes with trentepohlioid photobiont belong to the *Ostropomycetidae* subclass [13]. The *Ostropomycetidae* subclass encompasses nine orders, 34 families, 237 genera, and 4810 species according to current phylogenetically informed taxonomy [14]. This taxonomic group of *Ostropomycetidae* has emerged as a prolific source of bioactive natural products, exemplified by the baeomycesic acid from *Baeomyces* spp. [15], and depsidone and depside derivatives [16]. Recent investigations of *Graphis* mycobionts revealed antimicrobial diphenyl ether red pigment graphinone A from *Graphis* cf. *handelii* Zahlbr. with efficacy against methicillin-resistant *Staphylococcus aureus* [17]. These active substances have increasingly drawn widespread attention. However, the systematic characterization of compounds and bioactivities in *Ostropomycetidae* remains fragmented. According to the databases of PubMed, Web of Science, and CNKI, we comprehensively expanded the phytochemical research from 1935 to 2024, identifying 202 structurally diverse compounds from only 14 studied families in *Ostropomycetidae*. The maximum likelihood phylogenetic tree was constructed based on the sequences of ITS, mtSSU, and nuLSU of this group (Figure 1a). Morphological characteristics of the lichen thallus of some species in *Ostropomycetidae* were illustrated (Figure 1b). The lichenized fungi of *Ostropomycetidae*, a natural source producing a number of valuable compounds, are economically not feasible and profitable due to their slow growth. Their mycobiont cultures are the alternative sources that have become highly attractive for chemists recently. Out of 202 compounds, about half of these metabolites belong to phenols and their derivatives (including 28 phenolic compounds, 34 depsides, 10 tridepsides, and 22 depsidones), polyketides (including seven lactones, 17 phthalides and dibenzofurans, five macrolides, 23 chromones, 16 xanthones, and three others), nine fatty acids, 21 sesquiterpenes, four sterols, as well as three non-ribosomal peptides. The names and producers of these compounds are given in the following content. These compounds are mainly categorized into six classes: phenolic derivatives, polyketides, fatty acids, terpenoids, sterols, and non-ribosomal peptides, and they exhibit multifaceted bioactivities including antitumor, anti-inflammatory, antimicrobial, antiviral, and antioxidant properties, and so on, which underscores the pharmaceutical potential of them (Figure 2a,c). The aim of this review is to enhance our insight into the underexplored reservoir of bioactive compounds produced in the species of lichenized fungi *Ostropomycetidae* and to facilitate the therapeutic potential of understudied lichen species for novel drug discovery and utilization in the future.

## 2. Phenolic Compounds and Bioactivities

### 2.1. Phenols

**Figure 3:**
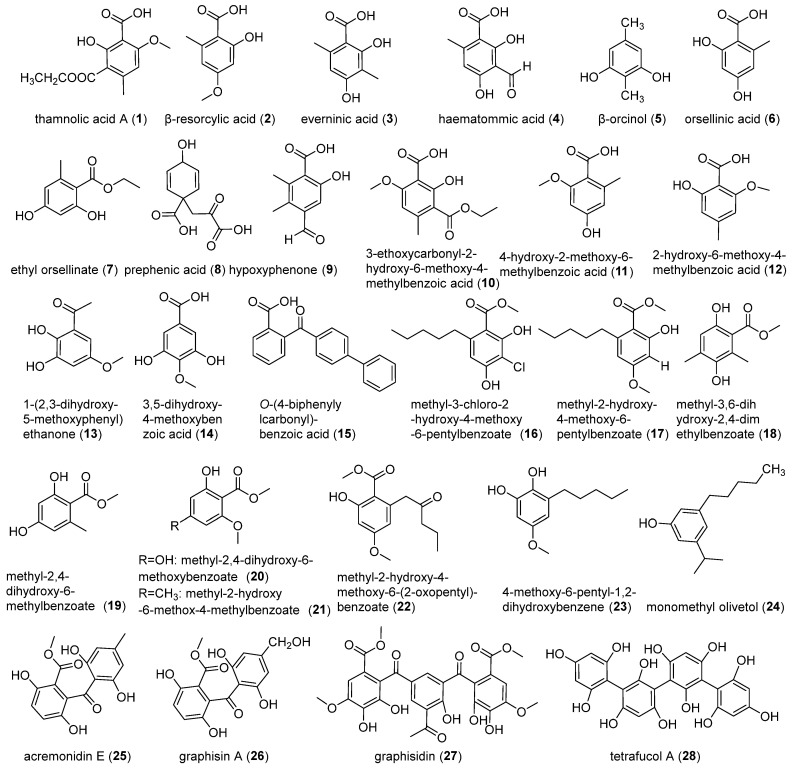
Chemical structures of phenol compounds (**1**–**28**).

Phenolic compounds represent the predominant secondary metabolites in lichenized fungi of the *Ostropomycetidae* subclass and exhibit special chemical diversity. The current study has documented 28 different phenolic derivatives within the subclass, which are systematically grouped into monophenols, diphenols, triphenols, and tetraphenols (Figure 3).

#### 2.1.1. Monophenol Derivatives

The chloroform extracts of *Thamnolia vermicularis* (Sw.) Schaer. yielded thamnolic acid A (**1**), *β-*resorcylic acid (**2**), and everninic acid (**3**) [18], while recent work on the Arctic lichen *Anamylopsora pakistanica* Usman & Khalid (*Baeomycetaceae*) found haematommic acid (**4**) featuring a unique 2,4-dihydroxy-6-pentylbenzoate scaffold [19]. Structure–activity relationship studies identified significant pharmacological properties. Compound **1** exhibited antibacterial activity [20], whereas compound **4** demonstrated good antitumour activity [19].

Orsellinic acid (2,4-dihydroxy-6-methylbenzoic acid) and its structural analogs have emerged as versatile synthetic precursors in pharmaceutical chemistry, biosynthesized across diverse taxa, including plants, lichenized fungi, and bacteria. The *β*-orcinol (**5**), a methylated analog of orcinol (3,5-dihydroxytoluene), serves as a crucial scaffold in bioactive compound development. Its natural occurrence was first documented in *Ochrolechia turneri* (Sm.) Zopf [18], with subsequent identification in *T. vermicularis* [21]. Orsellinic acid (**6**) was initially characterized in *Diploschistes muscorum* (Scop.) R. Sant. [22], and was later confirmed in *D*. *scrupossus*, *D*. *norman*, *D*. *diacapsis* [23], and *Ochrolechia frigida* (Sw.) Lynge [24]. Structural diversification of this core scaffold has yielded derivatives including ethyl orsellinate (**7**) from *G. handelii* mycobionts [17], along with prephenic acid (**8**) and hypoxyphenone (**9**) from *O. frigida* [24]. Compounds (**6**, **8**–**9**) demonstrated significant antioxidant activity [24]. Recent advances have uncovered novel orsellinic acid analogs in *T*. *vermicularis*, including 3-ethoxycarbonyl-2-hydroxy-6-methoxy-4-methylbenzoic acid (**10**), 4-hydroxy-2-methoxy-6-methylbenzoic acid (**11**), 2-hydroxy-6-methoxy-4-methylbenzoic acid (**12**), and 1-(2,3-dihydroxy-5-methoxyphenyl) ethanone (**13**). All of these compounds exhibited potent anti-amyloidogenic activity, inhibiting hen egg white lysozyme (HEWL) fibril formation with IC_50_ values of 0.11 μmol/mL and 0.10 μmol/mL, which suggested the therapeutic potential against protein-misfolding disorders [25].

Orsellinic novel biphenyl derivatives of 3,5-dihydroxy-4-methoxybenzoic acid (**14**) and *O*-(4-biphenylylcarbonyl)-benzonic acid (**15**) emerged from *D*. *pruinosum* and *O*. *frigida* [24,26]. Antarctic lichen *Pertusaria dactylina* (Ach.) Nyl. (*Pertusariaceae*) was found to biosynthesize a series of branched-chain methyl benzoates (**16**–**24**) featuring distinctive pentyl/methoxy substitutions. This chemically diverse series includes the following: chlorinated derivative of methyl-3-chloro-2-hydroxy-4-methoxy-6-pentylbenzoate (**16**), oxygenated analog of methyl-2-hydroxy-4-methoxy-6-pentylbenzoate (**17**), polyhydroxylated structure of methyl-3,6-dihydroxy-2,4-dimethylbenzoate (**18**), variably substituted isomers with distinct hydroxyl/methoxy position of methyl-2,4-dihydroxy-6-methylbenzoate (**19**), methyl-2,4-dihydroxy-6-methoxybenzoate (**20**), methyl-2-hydroxy-6-methoxy-4-methylbenzoate (**21**), methyl-2-hydroxy-4-methoxy-6-(2-oxopentyl)-benzoate (**22**), biphenyl precursor of 4-methoxy-6-pentyl-1,2-dihydroxybenzene (**23**), and olivetol derivative of monomethyl olivetol (**24**) [27,28]. Notably, only the chlorinated analog compound **16** demonstrated measurable antifungal activity against *Candida albicans*, showing moderate inhibition with an IC_50_ value of 246 ± 26 μmol/L [27]. This structure–activity correlation suggests that chlorine substitution at C-3 may play a key role in the antifungal activity.

#### 2.1.2. Polyhydroxylated Derivatives

The genus *Graphis* (*Graphidaceae*) serves as a prolific source of structurally intricate polyphenolic metabolites. Pioneering studies identified two architecturally complex diphenolic derivatives: acremonidin E (**25**) and its analog graphisin A (**26**), characterized by a unique 2,4-dihydroxy-6-pentylbenzoate scaffold [29]. Subsequent research revealed graphisidin (**27**), a triphenolic compound distinguished by dual methoxycarbonyl substitutions at C-3 and C-5 positions [30]. Only compound **25** exhibited significant pharmacological potential, showing dual bioactivities of antitubercular efficacy with MIC values of 0.157 μmol/mL and cytotoxic effects against breast cancer, oral epidermoid carcinoma KB, and small cell lung cancer cell line NCI-H187 with IC_50_ values ranging from 0.04–0.085 μmol/mL [29]. Notably, *O. frigida* yielded tetrafucol A (**28**), which is a tetraphenolic derivative representing one oxidized aromatic scaffold with antioxidant activity [24].

### 2.2. Depsides and Their Derivatives

**Figure 4:**
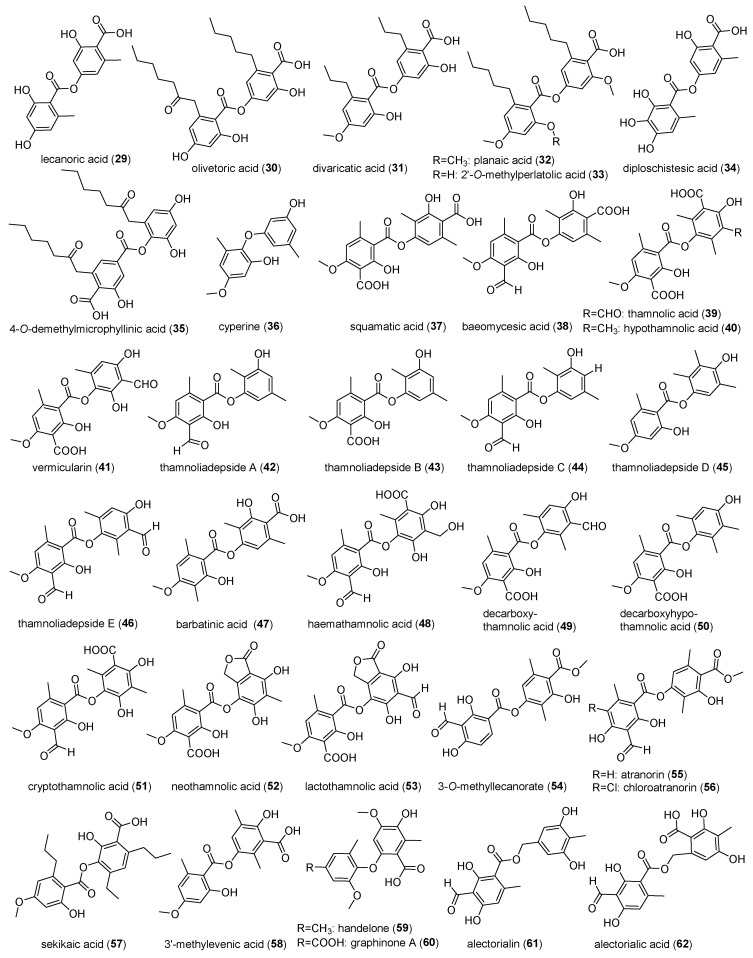
Chemical structures of depside compounds (**29**–**62**).

Depsides represent a structurally distinctive class of lichen phenolics. Lichen depsides are classified into three main groups based on the presence or absence of a C-3 alkyl substituent on their aromatic rings: orcinol-type—aromatic rings of both subunits lack a C-3 substituent; *β*-orcinol-type—aromatic rings of both subunits possess a C-3 substituent; and a hybrid-type—only one aromatic ring bears a C-3 substituent. While biosynthetically characteristic of lichen symbionts, these compounds demonstrate evolutionary convergence through their presence in select angiosperms and non-lichenized Ascomycota [31]. The researches on the pharmacological activity of depsides from *Ostropomycetidae* lichens has been acquired in the past decades [32]. Recent investigations confirm that *Ostropomycetidae* lichens remain the predominant producers of depsides, with a total of 34 structurally characterized compounds, including eight orcinol-type depsides, 20 *β*-orcinol-type depsides, four hybrid-type depsides, and two *benzyldepsides* (Figure 4).

#### 2.2.1. Orcinol-Type Depsides

Orcinol-type depsides are biosynthesized through exclusive oxidative coupling of orcinol (5-methylresorcinol) monomers. The archetypal member lecanoric acid (**29**), featuring a *para*-*para*’ ester linkage, was first characterized from the crustose lichen *Siphula ceratites* (Wahlenb.) Fr. (*Icmadophilaceae*) [33]. Subsequent chemotaxonomic studies have mapped its broad phylogenetic distribution across *Ostropomycetidae*, including *Ochrolechia parella* (L.) A. Massal. [34,35], *Ochrolechia androgyna* (Hoffm.) Arnold [36], *Pertusaria mccroryae* Björk, Goward & T. Sprib. [37], and *Lobothallia alphoplaca* (Wahlenb.) Hafellner [38]. Recent advances extended its detection to *D. muscorum* and *Placopsis contortuplicata* I.M. Lamb [22], and four *Diploschistes* species (*D. scrupossus*, *D. cinereocaesius*, *D. norman*, and *D. diacapsis*) [23]. Compound **29** exhibits dual pharmacological properties: broad-spectrum antifungal efficacy against clinically relevant strains [36] and potent antioxidant capacity via DPPH radical scavenging pattern [24,38]. Structural modifications and derivatives of compound **29** have been identified from the *G. handelii* mycobiont [17].

Olivetoric acid (**30**), first reported as a monophenolic secondary metabolite from crustose lichens *Ochrolechia* spp. (*Ochrolechiaceae*, *Ostropomycetidae*) [39], demonstrated dose-dependent anti-angiogenic effects through dual mechanisms: inhibition of rat adipose-derived endothelial cell proliferation and suppression of capillary-like tube formation in vitro [40]. Subsequent studies revealed its differential cytotoxicity against neural cells, showing IC_50_ values of 0.354 mmol/L in primary rat cerebral cortex cells versus 0.078 mmol/L in U87MG glioblastoma cell lines [41]. Divaricatic acid (**31**), another representative of this class, was initially isolated from *Pertusaria* spp. [42] and demonstrated exceptional antifungal potency against seven phytopathogens, including *Fusarium oxysporum* TR4 [43]. The structurally related compounds of planaic acid (**32**) and 2′-*O*-methylperlatolic acid (**33**) were co-isolated from *Pertusaria paramerae* A. Crespo & Vězda, which were differentiated by C-2′ methylation in the former [44]. Diploschistesic acid (**34**) has been identified to be ubiquitous in *Diploschistes* taxa, including *D. muscorum* [22], *D. diacapsis*, *D. scrupossus*, and *D. norman* [23]. 4-*O*-demethylmicrophyllinic acid (**35**) was isolated from *Ochrolechia* sp. [39]. The benzyl ether derivative of cyperine (**36**), as one phytotoxic metabolite, has been identified with antioxidant activity from *P. contortuplicata* [24].

#### 2.2.2. *β*-Orcinol-Type Depsides

This subclass comprises depsides biosynthesized through oxidative coupling of *β*-orcinol precursors. The prototype compound squamatic acid (**37**), featuring a characteristic ortho-para’ ester linkage, was first isolated from *Siphula* sp. [45]. Chemotaxonomic studies subsequently identified its occurrence in *Baeomyces rufus* (Huds.) Rebent. and *Baeomyces speciosus* (Körb. Ex Stein) Lindau [16], and in *T. vermicularis* mycobionts [18,46,47], and *Thamnolia subuliformis* (Ehrh.) W.L. Culb. [20]. Baeomycesic acid (**38**), the predominant depside in lichen systems, along with thamnolic acid (**39**), was initially isolated from lichen *Siphula* spp. [48]. Compounds (**37**–**38**) had antimicrobial efficacy to inhibit methicillin-resistant *S. aureus* [20]. Compound **38** also possesses more extensive bioactivities. It exhibited anti-inflammatory activity [47] and showed dose-dependent inhibitory effects on 5-lipoxygenase with an IC_50_ value of 8.311 μmol/L [49] and weak inhibitory activity on platelet-type 12(S)-lipoxygenase using a cell-based in vitro system in human platelets at a concentration of 267 μmol/L [50]. It exhibited a moderate radical scavenging antioxidant activity with an IC_50_ value of 1.608 mmol/L and good cytotoxic activity at the human epithelial carcinoma cell line Hela S3 with an IC_50_ value of 361 μmol/L [15]. In addition, compound **37** has other multitarget activities such as NFKB inhibition, antioxidant, lipoxygenase inhibition, glucosidase inhibition, and estrogen receptor agonism properties [51]. Thamnolic acid (**39**) has been identified in *Pertusaria corallina* (L.) Arnold [52] and *Graphis cincta* (Pers.) Aptroot [53] with general antibacterial activity [52].

Hypothamnolic acid (**40**), a structural analog of baeomycesic acid, has been identified in multiple taxa, including *Siphula* sp. [45], *Pertusaria* sp. [54], and *T. vermicularis* [47]. Chemotaxonomic studies reveal that the *Thamnolia* genus produces at least five specialized metabolites. Notably, vermicularin (**41**) has been consistently isolated from *T. vermicularis* across multiple studies [18,25,55], while thamnoliadepsides A–E (**42**–**46**) and barbatinic acid (**47**) were identified in *T. vermicularis*, and both exhibit anti-inflammatory activity characterized in the same species [18,47]. Mechanistic investigations demonstrate that compound **40** reduces intracellular reactive oxygen species (ROS) levels in hydrogen-peroxide-induced fibroblasts, suggesting potential cosmeceutical applications [55]. Furthermore, comparative bioactivity assays indicate that compounds (**38**, **41**) inhibit hen egg-white lysozyme (HEWL) fibril formation with IC_50_ values of 0.051 μmol/mL and 0.048 μmol/mL, respectively, highlighting their therapeutic potential against neurodegenerative pathologies [25]. Compounds (**41**–**42**) reveal differential antitumor activity with a growth inhibitory effect in prostate cancer cells with IG_50_ values of 70.06 µmol/mL and 79.37 µmol/mL, respectively [18].

Haemathamnolic acid (**48**), featuring a rare monoprotonated aromatic system, was isolated from *Pertusaria rhodesiaca* Vain. [56]. Decarboxythamnolic acid (**49**) co-occurred in *Pertusaria* spp. and *Siphula* spp. [48,54]. Decarboxyhypothamnolic acid (**50**) and cryptothamnolic acid (**51**) from *Pertusaria*, as well as neothamnolic acid (**52**) and lactothamnolic acid (**53**) from *Siphula ramalinoides* Nyl., further exemplified the structural diversification within this type of compound [54]. Moreover, the methylated derivative of 3-*O*-methyllecanorate (**54**) was identified in *T. vermicularis* [47]. Atranorin (**55**), a ubiquitously distributed lichen metabolite first characterized in *Siphula* spp. [48], occurs taxonomically across *Aspicilia* spp. [57], *Baeomyces* spp. [16], *O. parella* [35], *P. paramerae* [44], *T. vermicularis* [47], and *L. alphoplaca* [38]. And halogenated analogs of chloroatranorin (**56**) were isolated from *Siphula* sp. [48]. Compound **55** appeared to have anti-herbivore activity in observed slug–lichen interactions [57] and exhibited non-specific antioxidant effects as broad-spectrum antioxidants [38]. It also selectively inhibited breast cancer cell lines of MDA MB-231 and MCF-7 with IC_50_ values of 5.36  ±  0.85 µmol/mL and 7.55  ±  1.2 µmol/L, respectively [58], and showed an inhibitory effect on nitric oxide levels in lipopolysaccharide-stimulated macrophages and a high anti-inflammatory potential (75.99% at 0.067 μmol/mL) [59].

#### 2.2.3. Hybrid-Type Depsides

Hybrid depsides, characterized by the covalent integration of both orcinol and *β*-orcinol subunits, exhibit enhanced structural and functional diversity. Sekikaic acid (**57**), the inaugural hybrid *β*-orcinol/orcinol type depside, was isolated from *S. ceratites* [60] and subsequently identified in *L. alphoplaca* [38]. Compound **57** demonstrates multifaceted bioactivities, including antitumor activity and antibacterial properties [61], anti-diabetic and antioxidant potentials [62], and 1,1-diphenyl-2-picryl-hydrazil (DPPH) radical scavenging activity [63]. It also showed potent inhibition towards a recombinant strain of respiratory syncytial virus with an IC_50_ value of 0.014 μmol/mL and the respiratory syncytial virus A2 strain with an IC_50_ value of 0.018 μmol/mL [64], and significant α-glucosidase with IC_50_ values ranging from 7.9 to 149 μmol/L [65]. The methylated derivative 3′-methylevenic acid (**58**) was identified in *T. vermicularis* [47]. Three benzyl ether derivatives have been identified, including phytotoxic metabolite handelone (**59**) and graphinone A (**60**) from *G. handelii* [17,66]. Compound **59** showed dual antiviral activity, including anti-SARS-CoV-2 main protease activity with an IC_50_ value of 5.2 μmol/L and inhibition of HIV at a final concentration of 10 μmol/L [66].

#### 2.2.4. Benzyldepsides

In lichens, benzyldepsides occur much less often than depsides and depsidones. The benzyldepsides of alectorialin (**61**) and alectorialic acid (**62**), featuring a unique benzyl-bridged biaryl scaffold, were isolated from arctic lichen *A*. *pakistanica*. The former is a decarboxylated product of compound **62**. It also does not occur so often in lichens and is not widely distributed. Compound **62** demonstrates selective cytotoxicity against various cancer cell lines, including HeLa, HCT116, MDA-MB-231, and MRC6 [19].

### 2.3. Tridepsides

**Figure 5:**
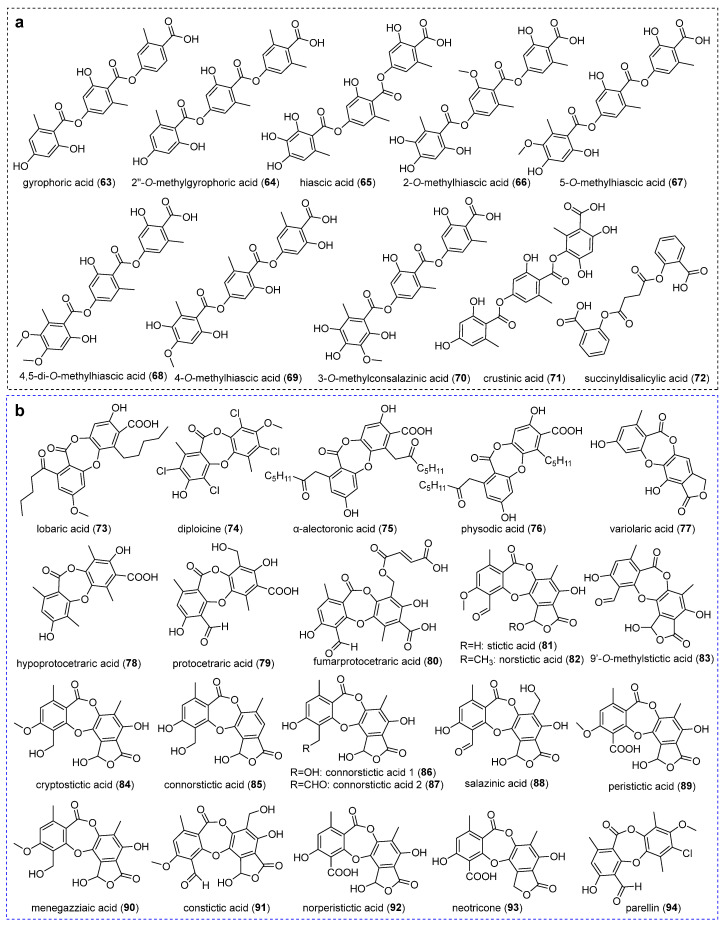
Chemical structures of compounds (**63**–**94**). (**a**) Tridepsides. (**b**) Depsidones.

Tridepsides, as fully oxidized polyketides, represent a chemically distinctive class of lichen metabolites with over a century of research history. Recent pharmacological investigations have validated their role as defensive lichenochemicals exhibiting multifunctional bioactivities. Current taxonomic analyses document tridepside production across 37 lichen families (spanning 111 genera and 526 species), with Parmeliaceae, Lobariaceae, and Peltigeraceae accounting for 68% of characterized compounds [67]. Notably, only ten tridepsides have been structurally elucidated from Ostropomycetidae lichens (Figure 5a), among which gyrophoric acid (**63**), hiascic acid (**65**), and 5-O-methylhiascic acid (**68**) demonstrate the broadest phylogenetic distribution.

The archetypal tridepside gyrophoric acid (**63**) was first isolated from *Ochrolechia* sp. [39], which was subsequently identified from *P. mccroryae*, *O. androgyna*, *D. diacapsis*, and *A. pakistanica* [19,23,37]. Compound **63** exhibits distinct pharmacological properties, including anticancer, wound healing, photoprotection, anti-aging, antioxidant, cardiovascular effect, DNA interaction, anti-diabetes, anti-Alzheimer’s, antibacterial, and antifungal [67]. It has minimum inhibitory concentration values ranging from 0.041 μmol/mL to 2.669 μmol/mL against the human colon carcinoma cell line LS174, human lung carcinoma cell line A549, malignant melanoma cell line Fem-x, chronic myelogeneous leukemia cell line K562 [68], melanoma cancer cells [69], anti-proliferative activity against human cervix carcinoma HeLa cell [70], and breast cancer cell MCF-7 [71]. On the mechanism, compound **63** impinges on topoisomerase 1 to cause cell cycle arrest, inhibits cell survival, and promotes apoptosis, so it has been an effective anticancer candidate drug [72]. Compound **63** also inhibited Protein Tyrosine Phosphatase 1B (PTP1B) activity with 50% inhibitory concentration values of 3.6 +/− 0.04 μmol/L in a non-competitive manner, and can be used for treating type 2 diabetes and obesity [73]. It exhibits the largest free radical scavenging activity of 2,2-diphenyl-1-picrylhydrazyl (DPPH) with an IC_50_ value of 105.75 μmol/L [68] and can be considered an important natural compound with potent anti-aging strategies in the field of cosmetics and healthcare [74]. Compound **63** also has the potential to inhibit SARS-CoV-2 Mpro and act as a lead compound for the development of antiviral drug candidates against SARS-CoV-2 [75].

Methylation-driven structural diversification generates specialized derivatives. Gyrophoric acid’s methylated derivative, 2′’-*O*-methylgyrophoric acid (**64**), was specifically isolated from *Diploschistes gyrophoricus* Lumbsch & Elix [76]. Hiascic acid (**65**) and its positional isomers demonstrate distinct methylation patterns. 2-*O*-methylhiascic acid (**66**) was found in *D*. *diacapsis* by LC-MS/MS [23]. 5-*O*-methylhiascic acid (**67**) and 4,5-di-*O*-methylhiascic acid (**68**) were characterized in *Ochrolechia* spp. [39], along with compound **67,** which was additionally found in *P. mccroryae* and *Rimularia* aff. *furvella* (Nyl. ex Mudd) Hertel & Rambold [37]. 4-*O*-methylhiascic acid (**69**) was identified in *G*. *handelii* [17]. The other specialized derivatives were isolated, including 3-*O*-methylconsalazinic acid (**70**) from *P. mccroryae* [37] and crustinic acid (**71**) and succinyldisalicylic acid (**72**) from *P. contortuplicata* with antioxidant activity [24].

### 2.4. Depsidones and Bioactivities

Depsidones, a class of polyphenolic polyketides, are structurally defined by an ether-bridge tricyclic system comprising two aromatic rings linked via a C-7-carbonyl-containing third ring [77]. These lichenized fungal metabolites exhibit remarkable structural diversity and pharmacological potential, particularly as scaffolds for multitarget therapeutic agents [78]. To date, 22 depsidones have been characterized from *Ostropomycetidae* lichens (Figure 5b).

#### 2.4.1. Orcinol-Type Depsidones

The other remaining key depsidone derivatives were successively characterized, including lobaric acid (**73**) from *T. vermicularis* [50] and *L*. *alphoplaca* [38], diploicine (**74**) and α-alectoronic acid (**75**) from *O. parella* and *Aspicilia radiosa* [34,79], α-alectoronic acid (**75**) from *O. parella* [35], and physodic acid (**76**) from *D. diacapsis* [23]. Notably, these derivatives exhibit distinct pharmacological profiles. Compound **73** demonstrated multiple bioactivities, including antibacterial and antioxidant [80], scavenging free radicals [38], and inhibiting the ability of platelet-type 12(S)-lipoxygenase with an IC_50_ value of 28.5 mmol/L [50]. Compound **75** exhibited antitumor activity against the B16 murine melanoma cell line with an IC_50_ value of 10.3 µmol/L [35] and moderate inhibition of recombinant Plk1 kinase with an IC_50_ value of 1.7 µmol/L [81]. Compound **76** showed anti-inflammatory activity as potent inhibitors of mPGES-1 with an IC_50_ value of 0.4 µmol/L [82], higher anti-proliferative activity with an IC_50_ value of 171 µmol/L [83], and cytotoxic, apoptotic, and cell migration inhibitory effects on lung adenocarcinoma A549 cell line [84], and both LNCaP and DU-145 cell lines [85]. Variolaric acid (**77**) was extracted from both *O. parella* and *A. radiosa*, displaying antioxidant properties [79], antitumor activity against murine cancer cell line B16-F1 with an IC_50_ value of 38.7 µmol/L [35], and antiviral drug candidates against SARS-CoV-2 main protease inhibition [75]. 

#### 2.4.2. *β*-Orcinol-Type Depsidones

Hypoprotocetraric acid (**78**), the first isolated from *Siphula* sp. in *Ostropomycetidae* [45] , was subsequently identified in *Phaeographis* sp. [86]. Structural analogues of protocetraric acid (**79**) and fumarprotocetraric acid (**80**) were present in *O. androgyna* [36]. Compound **79** demonstrated broad-spectrum efficacy against Gram-positive pathogens (*Bacillus subtilis* and *S. aureus*), Gram-negative pathogens (*Enterobacter cloaceae*, *Escherichia coli*, *Klebsiella pneumoniae*, and *Botrytis cinerea*) [36], *Salmonella typhi*, and the fungus *Trichophyton rubrum* [87]. It also showed moderate inhibitory antitubercular activity [88], strong anticancer activity toward human melanoma FemX cell lines and the human colon carcinoma LS174 cell line with IC_50_ values ranging from 0.953 μmol/mL to 0.161 μmol/mL [89], and possessed stronger alpha-glucosidase inhibitory activity with IC_50_ values ranging from 43.7 to 110.1 μmol/L [90]. Compound **80** exhibited broad-spectrum activity against Gram-positive bacteria (*Bacillus mycoides*, *B. subtilis*, and *S. aureus*) and Gram-negative pathogens (*E. cloaceae*, *E. coli*, and *K. pneumoniae*, with MIC value as low as 0.066 μmol/mL). It was also observed against pathogenic fungi, including *Mucor mucedo*, *Aspergillus flavus*, and *C. albicans* with MIC values ranging from 0.324 to 0.529 μmol/mL [36], and possessed the expectorant and antioxidant properties [91,92].

Stictic acid (**81**) was initially identified in *Huilia albocaerulescens* (Wulfen) Hertel [57], *Baeomyces* spp. [16], *G. desquamescens* [93], and *O. androgyna* [36]. Subsequent studies have expanded its taxonomic distribution to include *Coccotrema hahriae* T. Sprib. & Tønsberg [37], *Pertusaria* spp. [37,53,94], *Aspicilia goettweigensis* (Zahlbr.) Hue [95], *Leucodecton occultum* (Eschw.) Frisch [53], *D*. *norman* [23], and *D. pruinosum* [26]. Stictic acid derivatives were successively identified; norstictic acid (**82**) was first isolated from *Siphula* spp. [48] and later detected in *Aspicilia* spp. [37,79,95], *C. hahriae* and *Phlyctis argena* (Spreng.) Flot. [37], *D*. *norman* [23], *D. pruinosum* [26], *Graphis* spp. [53,93], *L. occultum* [53], *O. parella* [80], and *Pertusaria pseudocorallina* (Lilj.) Arnold [34]. Compound **81** demonstrated moderate antimicrobial activity at MIC values ranging from 0.647 μmol/mL to 1.294 μmol/mL for bacteria and 2.589 μmol/mL for fungi, respectively [36]. Compound **81** also showed moderate anticancer activity with an IC_50_ value of 0.076 μmol/mL for the cell line HT-29 and a low growth inhibition on the nonmalignant cell line MRC-5 with an IC_50_ value of 6.415 μmol/mL [96]. Compounds (**81**–**82**) showed anti-herbivore activity [57] and significant alpha-glucosidase inhibition with IC_50_ values ranging from 10.4 to 130 µmol/L [97]. Compound **82** also exhibited activity against *Mycobacterium tuberculosis* with an MIC value of 0.161 μmol/mL and 168 µmol/L [88], and antioxidant activity [34].

The methylated derivatives include 9’-*O*-methylstictic acid (**83**) isolated from *D. pruinosum* [26] and cryptostictic acid (**84**) identified in *P. mccroryae*, *C*. *hahriae* [37], and *D. norman* [23]. The other derivatives of compound **81** have been further characterized. For example, connorstictic acid (**85**) was isolated from *C*. *hahriae* and *Aspicilia cinerea* (L.) Körb. [37], *P. pseudocorallina* [34], and *D. norman* [23]. Connorsticitic acid 1 (**86**), connorsticitic acid 2 (**87**), and salazinic acid (**88**) were isolated from *P. pseudocorallina* [98]. Peristictic acid (**89**), menegazziaic acid (**90**), constictic acid (**91**) and norperistictic acid (**92**) were isolated from *P*. *mccroryae*, *C*. *hahriae* [37], *Baeomyces* spp. [16] and *Phaeographis* sp. [86], respectively. These compounds demonstrate various pharmacological activities. Compound **88** exhibited significant apoptotic activities [99], strong anticancer activity with IC_50_ values ranging from 0.92 μmol/mL to 0.155 μmol/mL [89], significant intestinal alpha-glucosidase inhibitory activities with IC_50_ values of 4.17 ± 0.18 μmol/mL (*p* < 0.001) [100], and radical scavenging action with IC_50_ values of 312.14 µmol/L [101]. Compound **90** demonstrated radical scavenging activity [38]. Neotricone (**93**), a novel C-methylated derivative with hydrogen-bonded hydroxy groups with unique C-methyl/methylene groups and hydrogen-bonded hydroxy, was isolated from *Phaeographis* sp. [86]. And parellin (**94**) was isolated from *O. parella* [35].

## 3. Aliphatic Acids and Bioactivities

**Figure 6:**
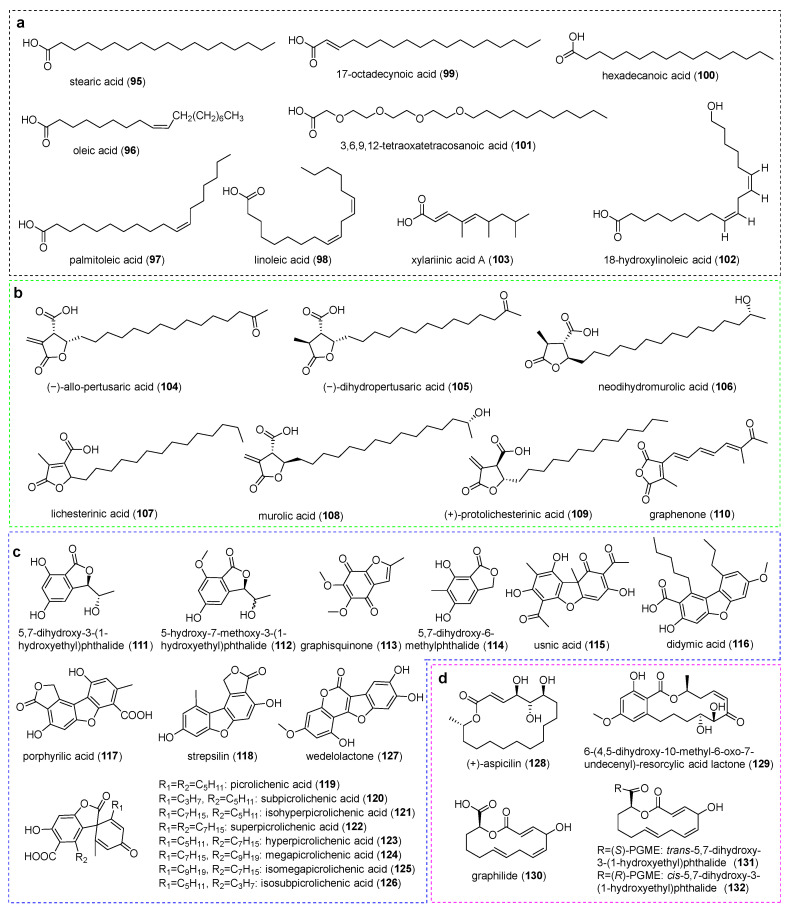
Chemical structures of compounds (**95**–**132**). (**a**) Aliphatic Acids. (**b**) Lactones. (**c**) Phthalides and dibenzofurans. (**d**) Macrolides.

The unique growth conditions of lichens promote the biosynthesis of aliphatic acids (fatty acids) with distinct concentration profiles and structural diversity. While lichen-derived fatty acids share carbon chain similarities with non-lichenized fungi (C16–C24), their biosynthesis primarily occurs via tricarboxylic acid (TCA) cycle modifications, resulting in lower structural complexity and abundance compared to plant systems [2]. To date, nine aliphatic acids have been characterized from *Ostropomycetidae* lichens (Figure 6a).

The common fatty acid derivatives of stearic acid (**95**), oleic acid (**96**), palmitoleic acid (**97**), and linoleic acid (**98**) were identified in *Aspicilia* spp. [102]. Stearic acid (**95**), linoleic acid (**98**), 17-octadecynoic acid (**99**), and hexadecanoic acid (**100**) were isolated from *Platygramme caesiopruinosa* (Fée) Fée [103]. Compound **97** showed therapeutic potential for chronic diseases, particularly cardiovascular disorders [104], and compound **98** exhibited multifunctional dermatological effects, including the repair of the skin barrier, wound healing acceleration, and anti-aging properties [105]. Compound **100** demonstrated concentration-dependent antimicrobial activity against human commensal and pathogen *S. aureus* [106]. In addition, 3,6,9,12-tetraoxapentacosanoic acid (**101**) and 18-hydroxylinoleic acid (**102**) were identified from *O. frigida*, both demonstrating antioxidant activity [24]. Xylarinic acid A (**103**) was discovered in *G. handelii* [66], exhibiting broad-spectrum antifungal activity against plant pathogenic fungi including *Pythium ultinum*, *Magnaporthe grisea*, *Aspergillus niger*, *Alternaria panax*, and *Fusarium oxysporium* [107].

## 4. Polyketides and Bioactivities

### 4.1. Lactone-Type Compounds

Aliphatic acids undergo enzymatic cyclization to form a γ-lactone characterized by a five-membered lactone ring. The key spectral features are reflected in both a diagnostic ^13^C NMR signal for the lactonized carbonyl carbon and moderate deshielding of the oxygen-adjacent ring carbon. Structural diversity arises from alkyl chain lengths (C11, C13, or C15), functional group substitutions (ketone, hydroxyl, terminal carboxyl, and acetoxy), as well as electronic effects. Exocyclic methylene groups induce greater shielding versus conjugated endocyclic double bonds [31]. To date, only seven γ-lactones have been identified in *Ostropomycetidae* lichens (Figure 6b). Among them, (−)-allo-pertusaric acid (**104**) and (−)-dihydropertusaric acid (**105**) were isolated from *Pertusaria albescen*s (Huds.) M. Choisy & Werner [108]. Additionally, neodihydromurolic acid (**106**), lichesterinic acid (**107**), murolic acid (**108**), and (+)-protolichesterinic acid (**109**) were identified from *Ochrolechia* spp. [39]. Graphenone (**110**), a compound exhibiting an orangish-yellow hue, was identified in *Graphis scripta* (L.) Ach. and *G. desquamescens* [93]. Compounds (**107** and **109**) exhibit potent antitrypanosomal activity against *Trypanosoma brucei* with MIC values of 12.5 µmol/L and 6.30 µmol/L, respectively [109]. Compound **109** also acted as the inhibitor of platelet-type 12(S)-lipoxygenase with an IC_50_ value of 28.5 mmol/L and 77.0 mmol/L [50], and had anti-proliferative activity against HeLa cells [110] and antitumor activity against HER2-overexpressing breast cancer [111].

### 4.2. Phthalides and Dibenzofurans

Phthalides constitute a structurally distinct class of natural products with restricted taxonomic distribution, primarily identified in higher plants and lichen-forming fungi. Mycophenolic acid analogues have emerged as pharmacologically significant representatives of this type of compound [112,113]. Seventeen phthalide derivatives have been isolated from *Ostropomycetidae* lichens (Figure 6c). These compounds include 5,7-dihydroxy-3-(1-hydroxyethyl)-phthalide (**111**) and 5-hydroxy-7-methoxy-3-(1-hydroxyethyl) phthalide (**112**) from *Graphis proserpens* Vain. [114] and graphisquinone (**113**) from *G*. *script*a and *G. desquamescens* [93]. Notably, 5,7-dihydroxy-6-methylphthalide (**114**), derived biosynthetically from alectorialic acid, was discovered in *A. pakistanica* with a good antitumour activity against the HeLa cell line from 0.311 ± 0.05 to 1.86 ± 0.155 μmol/mL, against the HCT116 cell line from 0.572 ± 0.089 to 1.099 ± 0.122 μmol/mL, against the MDA-MB-231 cell line from 0.599 ± 0.056 to 1.393 ± 0.155 μmol/mL, and against the MRC6 cell line from 1.038 ± 0.1 to 1.754 ± 0.161 μmol/mL, respectively [19].

Dibenzofurans feature two benzene rings fused to a central furan moiety (positions 2,3 and 4,5), exhibiting three primary structural variations including furan ring annulation patterns, isoprenyl substitutions, and aliphatic side chain modifications. These compounds exhibit diverse biological properties and were traditionally considered to originate primarily from lichens and ascomycetes [115]. Usnic acid (**115**), the most prominent dibenzofuran derivative, serves as a characteristic yellow pigment in lichens and displays broad pharmacological applications in dietary supplements and pharmaceuticals [113,116,117]. Usnic acid was first isolated from *B. rufus* and *B. speciosus* [16], and was then identified from *T. vermicularis* [118], *Aspicilia* sp. [102], and *A. pakistanica* [19]. Compound **115** exhibited broad antibiotic activity [47], antiviral activity [119], and antimicrobial properties and antitumor activity against cell lines with IC_50_ values of 0.37 μmol/mL [19]. Additional dibenzofuran derivatives include didymic acid (**116**) isolated from *Pertusaria flavens* Nyl. [53], porphyrilic acid (**117**) from *Siphula* spp. [48] and *Dibaeis* sp. [37], and strepsilin (**118**) from *Siphula* spp. [48]. Compound **116** was active against *S. aureus* with a minimum inhibitory concentration of 0.024 μmol/mL [120], and compound **117** exhibited noticeable inhibition of the 5-lipoxygenase enzyme and inhibition of xanthine oxidase enzyme with an IC_50_ value of 0.255 +/− 0.002 μmol/mL [121].

The genus *Pertusaria* produced a chemically related series of compounds, including picrolichenic acid (**119**), subpicrolichenic acid (**120**), and isosubpicrolichenic acid (**121**) from *P. amara* and *P. truncata* [122,123]. Compound **119** also occurred in *O. parella* [34]. Further derivatives, including superpicrolichenic acid (**122**), hyperpicrolichenic acid (**123**), megapicrolichenic acid (**124**), isomegapicrolichenic acid (**125**), and isosubpicrolichenic acid (**126**), were characterized in *P. truncata* [122,123]. Notably, wedelolactone (**127**), a pharmacologically active compound typically associated with Asteraceae plants, was recently identified in the lichen *O*. *frigida* with antioxidant activity [24]. Compound **127** demonstrated a broad spectrum of therapeutic potential, including anticancer, anti-inflammatory, anti-obesity, anti-myotoxic, antimicrobial, anti-diabetic, and tissue-protective activities [124]; moreover, the compound reduced the pathological damage of the liver, decreased ALT and AST, MMP, ROS, and inflammatory factors, and decreased hepatocyte viability in vitro [125].

### 4.3. Macrolides

Macrolides, a class of antimicrobial agents with broad clinical applications, have been identified in lichenized fungi of the *Ostropomycetidae* subclass, with five distinct representatives characterized to date (Figure 6c). Notably, (+)-aspicilin (**128**) with anti-herbivore activity was isolated from *Aspicilia gibbosa* (Ach.) Körb. and *A. cinerea* [57], and *Aspicilia contorta* (Hoffm.) Körb. [95]. While zearalenone derivatives are predominantly associated with *Fusarium* species, 6-(4,5-dihydroxy-10-methyl-6-oxo-7-undecenyl)-resorcylic acid lactone (**129**), which represents the first lichen-derived zearalenone analog, was isolated from both mycobiont cultures and intact thalli of *Baeomyces placophyllus* Ach.; this compound exhibits topoisomerase I and II inhibitory activities with an IC_50_ value of 0.275 μmol/mL and apoptosis producing activity on tumor cells with a value of 0.028 μmol/mL [126]. Three 14-membered macrolides were isolated from axenic cultures of *Graphis vestitoides* (Fink) Staiger, including graphilide (**130**), *trans*-5,7-dihydroxy-3-(1-hydroxyethyl)-phthalide (**131**), and *cis*-5,7-dihydroxy-3-(1-hydroxyethyl)-phthalide (**132**) [127].

### 4.4. Chromones

**Figure 7:**
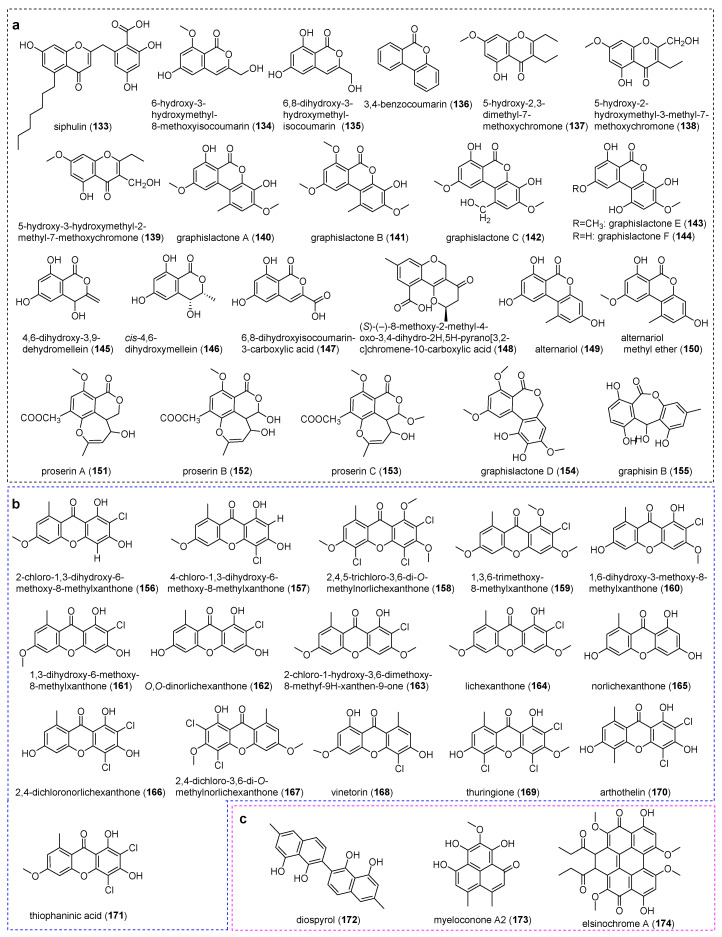
Chemical structures of compounds (**133**–**174**). (**a**) Chromones. (**b**) Xanthones. (**c**) Other polyketides.

Chromones and their derivatives have emerged as multitarget therapeutic candidates in recent decades [128]. To date, 23 chromones and their derivatives have been characterized in *Ostropomycetidae* lichens (Figure 7a). Siphulin (**133**), a chromone-type lichen chromone-like metabolite, was isolated from *S*. *ceratites* [33,60]. Both isocoumarin derivatives of 6-hydroxy-3-hydroxymethyl-8-methoxyisocoumarin (**134**) and 6,8-dihydroxy-3-hydroxymethylisocoumarin (**135**) were isolated from *G*. *proserpens* [114]. Compound **135** featured a 3,6,8-substituted isocoumarin framework [127] and exhibited moderate anti-HSV-1 and antimycobacterial activities with IC_50_ and MIC values of 0.24 μmol/mL and 0.12 μmol/mL, respectively [129]. 3,4-benzocoumarinaltern (**136**) was identified in *Pertusaria* sp. [130]. Three methylated chromones of 5-hydroxy-2,3-dimethyl-7-methoxychromone (**137**), 5-hydroxy-2-hydroxymethyl-3-methyl-7-methoxychromone (**138**), and 5-hydroxy-3-hydroxymethyl-2-methyl-7-methoxychromone (**139**) were isolated from *G*. *scripta* mycobiont cultures [131]. 

Graphislactones A–C (**140**–**142**) and graphislactones F (**144**) were isolated from *Graphis prunicola* Vain., graphislactones A and C were also found in *Graphis cognata* Müll. Arg., and *G. scripta*. Graphislactones E (**143**) was detected in *G*. *scripta* and *G. prunicola* [132]. Compound **140** exhibits cytotoxicity against human lung fibroblasts with a moderate activity towards acetylcholinesterase with an IC_50_ value of 0.027 μmol/mL [133], antioxidant capacity equivalent to ascorbic acid in hepatocytes, and anti-inflammatory response by lipopolysaccharide in macrophages [134]. Both mellein derivatives of 4,6-dihydroxy-3,9-dehydromellein (**145**) and *cis*-4,6-dihydroxymellein (**146**) were produced by *G*. *proserpens* [30], and the latter was also isolated from *G. vestitoides*. Compound **146** showed different degrees of phytotoxicity towards sunflower leaves and seedlings and may contribute to the severity of the sunflower disease caused by *Phomopsis helianthin* [135]. 6,8-dihydroxyisocoumarin-3-carboxylic acid (**147**) was isolated from crustose lichen *G. vestitoides* [127], and (S)-(–)-8-Methoxy-2-methyl-4-oxo-3,4-dihydro-2H,5H-pyrano[3,2-c] chromene-10-carboxylic acid (**148**) was characterized by methyl substitution and meta-coupled aromatic protons in *Graphis* sp. [30]. 

Alternariol (**149**), a mycotoxin with genotoxic properties, was found in *G. cognata* and *Pertusaria* sp. [132]. And alternariol’s methyl ether (**150**) was isolated from *Pertusaria* sp. [130]. Isocoumarin is one chemical compound with a lactonic α-pyranone ring fused to a benzene ring. Compounds (**149**–**150**) can cause potential acute and chronic human health problems as mycotoxins [136,137]. Proserins A–C (**151**–**153**), as the isocoumarin derivatives, were produced in *G*. *proserpens* [114], with proserin A also detected in *Graphis* spp. [30]. Graphislactone D (**154**) has been found in the lichen *G. prunicola* [132]. Graphisin B (**155**), a unique tetracyclic chromone, was identified in *Graphis tetralocularis* C. Bock & M. Hauck [29].

### 4.5. Xanthones

Xanthones, which are polyketide-derived secondary metabolites extensively studied in free-living fungi, have recently gained prominence in lichen chemistry. Their core structure arises from the linear condensation of seven acetate/malonate units with subsequent orsellinic acid-type cyclization to form two aromatic rings interconnected via ketonic carbonyl groups and ether-oxygen linkages. This unique biosynthetic pathway confers structural diversity and multitarget pharmacological potential, positioning lichen xanthones as valuable candidates for drug discovery [138]. A total of 16 xanthones have been characterized from *Ostropomycetidae* lichens (Figure 7b).

*Pertusaria sulphurata* Müll. Arg. yielded eight chlorinated compounds including 2-chloro-1,3-dihydroxy-6-methoxy-8-methylxant (**156**), 4-chloro-1,3-dihydroxy-6-methoxy-8-methylxanthone (**157**), 2,4,5-trichloro-3,6-di-*O*-methylnorlichexanthone (**158**), 1,3,6-trihydroxy-8-methylxanthone (**159**), 1,6-dihydroxy-3-methoxy-8-methylxanthone (**160**), 1,3-dihydroxy-6-methoxy-8-methylxanthone (**161**), *O*,*O*-dinorlichexanthone (**162**), and 2-chloro-1-hydroxy-3,6-dimethoxy-8-methyf-9H-xanthen-9-one (**163**) [139]. Lichexanthone (**164**) was identified from *Ochrolechia* sp., *Pertusaria wui* Q. Ren [39]. Norlichexanthone (**165**) was isolated from *Pertusaria laeviganda* Nyl. [140] and exhibited the strongest inhibitory activity against 16 cancer-related protein kinases, including aurora-B, PIM1, and VEGF-R2 with IC_50_ values ranging from 0.40 to 74.0 µmol/L, against these three protein kinases with IC_50_ values ranging from 0.3 to 11.7 µmol/L [141], and non-specific antioxidant effects with IC_50_ values greater than 200 µmol/L [140]. 2,4-dichloronorlichexanthone (**166**) was isolated from *P. paramerae* [44], which demonstrated antagonistic activities 2,4-dichloronorlichexanthone demonstrated antagonistic activities against *Streptococcus agalactiae* and *S. aureus* with MIC values of 10.21 µmol/L and 20.42 µmol/L, respectively [142]. and 2,4-dichloro-3,6-di-O-methylnorlichexanthone (**167**), vinetorin (**168**), and thuringione (**169**) were identified from *Pertusaria* sp. [143]. Compound **168** exhibited remarkable antagonistic activities against *Streptococcus agalactiae* and *S. aureus* with MIC values of 10.21 µmol/L and 20.42 µmol/L, respectively [142]. Arthothelin (**170**) was identified in *Pertusaria* sp. [108] and *Dibaeis* sp. [37]. Thiophaninic acid (**171**) has been isolated from *P. sulghuvata* [139] and *P. paramerae* [44].

### 4.6. Other Polyketides

Several other polyketide compounds have been identified from the *Ostropomycetidae* lichens (Figure 7c). Diospyrol (**172**), an anthelmintic drug from *Diospyros mollis* griff, was isolated from the lichen *O*. *frigida*, and it showed remarkable antioxidant activity [24]. Myeloconone A2 (**173**), as one new phenalenone of yellow pigment, was first identified from lichen *Myeloconis erumpens* P.M. McCarthy & Elix [144]. Another perylenequinone pigment, elsinochrome A (**174**), was identified in *Graphis elongata* Vain. mycobiont cultures. Elsinochromes, as the perylenequinone toxins from pathogenic fungi, are crucial mediators in fungal–host interactions [145]. And compound **174** displayed photodynamic effects against Gram-positive bacteria *Pseudomonas syringae* and fungus *Alternaria mali* [146].

## 5. Terpenoids and Bioactivities

Sesquiterpenoids, which are crucial components of plant chemical defense systems, exhibit selective bioactivity across antimicrobial, anticancer, and anti-inflammatory domains while maintaining low cytotoxicity toward normal cells [147]. Three structural classes have been characterized in *Ostropomycetidae* lichens: guaiane-type, sesquiterpene-quinones, and eremophilane-type derivatives.

**Figure 8:**
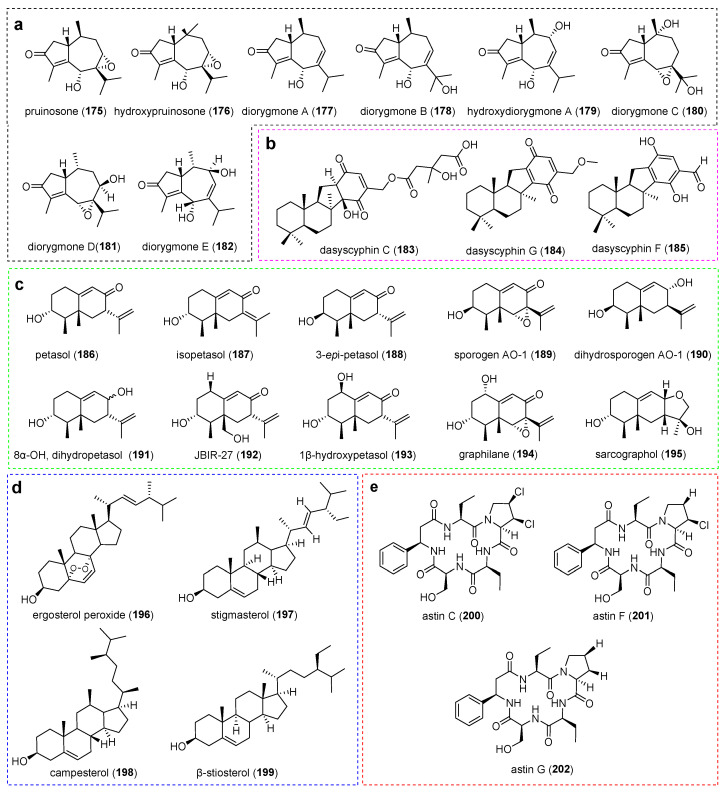
Chemical structures of compounds (**175**–**202**). (**a**) Guaiane-type sesquiterpenes. (**b**) Sesquiterpene quinones. (**c**) Eremophilane-type sesquiterpenes. (**d**) Sterols. (**e**) Non-ribosomal peptides.

Guaiane-type sesquiterpenes were characterized by a bicyclo[5.3.0] decane framework; these compounds demonstrate multifaceted pharmacological activities such as antitumor, anti-inflammatory, and antibacterial effects [148]. Six guaiane-type sesquiterpenes have been isolated from *Ostropomycetidae* lichens (Figure 8a). Pruinosone (**175**) and hydroxypruinosone (**176**) were isolated from *D. pruinosum*, both featuring a 2-methylcyclopent-2-en-1-one moiety. The former possessed antifungal activity and cytotoxic activity, and the latter has shown an inhibitory effect on fungi [149]. Diorygmones A–B (**177**–**178**) possessed a guaiane-type sesquiterpenoid core and were identified from *Diorygma* sp. mycobiont cultures with pruinosone (**175**) and hydroxydiorygmone A (**179**) [150,151]. Diorygmones C–E (**180**–**182**) were isolated with diorygmone B from *D. pruinosum* [26]. Compounds (**177**–**178**) showed moderate cytotoxic activity against the HepG2 cell line with IC_50_ values of 0.259 ± 0.009 and 0.136 ± 0.006 μmol/mL, respectively [151], and compounds (**178**, **180**–**181**) were inhibitory to *S. aureus* [26]. Compound **180** showed moderate α-glucosidase inhibition with an IC_50_ value of 160 ± 2.2 µmol/L and significant inhibitory activity toward nitric oxide production in LPS-stimulated RAW264.7 cells with an IC_50_ value of 8.11± 0.21 µmol/L [150].

Sesquiterpene quinones, hybrid metabolites combining a C15-sesquiterpenoid backbone with a C6-benzoquinone/quinol moiety, exhibit broad pharmacological potential including anticancer, anti-inflammatory, antimicrobial, antiviral, and fibrinolytic activities [152]. Three representatives have been characterized in *Stictidaceae* lichens (Figure 8b). Dasyscyphin C (**183**), dasyscyphin G (**184**), and dasyscyphin F (**185**) were isolated from the lichen *Stictidaceae*. Compound **183** possesses a 2-cyclohexene-1,4-dione ring [153]. Dasyscyphins showed a variety of biological activities. For example, compound **183** showed cytotoxic activities against human cell lines, including HepG2, Hela S3, U937, Colo-320, and Jurkat with IC_50_ values from 0.001 to 0.006 μmol/mL [154]. Compounds **183** and **186** have a good antitumor activity and are moderately active against the three cell lines of melanoma MDA-MB-435, breast MDA-MB-231, and ovarian OVCAR3 with IC_50_ values ranging from 4 to 16 mmol/L [153], and antimicrobial activity to *Pseudomonas aeruginosa*, *Methicillin resistant*, *S. aureus*, and *Bacillus anthraci* [153]. Compound **183** showed good leishmanicidal activity at 1.982 μmol/mL concentration with an IC_50_ value of 0.892 μmol/mL against *Leishmania major* promastigote [155].

Eremophilane sesquiterpenes, characterized by a 6,7-seco-eudesmane skeleton, demonstrate phytotoxic and immunomodulatory properties across both plants and fungi [156]. Nine derivatives were isolated from *Sarcographa tricosa* (Ach.) Müll. Arg. mycobiont cultures (Figure 8c). The eremophilane compounds, including petasol (**186**), isopetasol (**187**), 3-*epi*-petasol (**188**), sporogen AO-1 (**189**), dihydrosporogen AO-1 (**190**), 8α-OH, dihydropetasol (**191**), JBIR-27 (**192**), 1*β*-hydroxypetasol (**193**), and sarcographol (**195**), were characterized from *S. tricosa* mycobiont cultures [157]. Compounds **189**–**190** and graphilane (**194**) were additionally detected in *Graphis* sp. mycobiont axenic cultures [158]. Compounds **186** and **189** exhibited cytotoxicity to HeLa cells and robust growth-restoring activity of *Saccharomyces cerevisiae* mutant strain [159]; the latter also showed cytotoxicity against human cervical carcinoma cell line HeLa at IC_50_ values of 8.3 μmol/L [160]. Compounds **189** and **190** caused significant inhibition of radicle growth against *Amaranthus hypochondriacus* and *Echinochloa crus-galli* at IC_50_ values of 0.17 mmol/L and 0.30 mmol/L, respectively [161]. Compound **194** showed moderate cytotoxic activity against the K562 cancer cell line with an IC_50_ value of 87.20 ± 0.76 μmol/L [158].

## 6. Steroids and Bioactivities

Sterols, which are essential eukaryotic membrane components, regulate cellular permeability and signaling processes. Phytosterols such as stigmasterol, campesterol, and *β*-sitosterol are distinguished by C-24 alkyl substituents (methyl for campesterol; ethyl for *β*-sitosterol) on their side chains [162]. Four steroidal compounds have been characterized from *Ostropomycetidae* lichens (Figure 8d), with ergosterol derivatives being predominant.

Ergosterol peroxide (**196**), a C28-sterol widely documented in medicinal mushrooms, was identified in *O*. *parella* [35] and *S. tricosa* [157], and showed anti-inflammatory activity with an IC_50_ value of 0.016 μmol/mL [163], antimicrobial activity, cytotoxic effects against a wide range of cells, immunosuppressive and anti-inflammatory activities, antiatherosclerotic effects, antifibrotic effects, insecticidal and phytotoxic effects, and cytotoxic effects against different tumor cell lines including MT-1 breast cancer cells, human intrahepatic cholangiocarcinoma cells (HuCCA-1), HeLa cells, prostate cells, colon carcinoma cells, and the myeloma cell line U266 [164]. Three key sterols have been characterized in *Ostropomycetidae* lichens with stigmasterol (**197**) and campesterol (**198**) from *Aspicilia* sp. [102] and *β*-Sitosterol (**199**) from *T. vermicularis* [55] and *D. pruinosum* [26]. Stigmasterol (**197**), biosynthesized through the mevalonate pathway, shares structural homology with β-sitosterol but is distinguished by a *trans*-configured double bond in its side chain [165]. This phytosterol has garnered significant pharmacological attention for its multifaceted bioactivities, including anti-inflammation, anti-diabetes, antioxidization, lowering blood cholesterol, and antitumor bioactivity (e.g., breast, lung, liver, and ovarian cancers) [166]. Compound **197** indicated potent pharmacological effects such as anticancer, anti-osteoarthritis, anti-inflammatory, anti-diabetic, immunomodulatory, antiparasitic, antifungal, antibacterial, antioxidant, and neuroprotective properties [167]. It also showed pathogen infection and abiotic stress, including cold, salinization, drought, UV radiation, heavy metals, and stress phytohormones in plants [165]. Compounds **198** and **199** exhibited strong antimicrobial activity against pathogenic microbes including *B*. *subtilis*, *S*. *pyogenes*, *S*. *aureus*, *K*. *pneumoniae*, *E*. *coli*, *P*. *aeruginosa*, *S*. *typhii*, *A*. *fumigatus*, *C*. *albicans*, *C*. *krusei*, and *A*. *niger* [168]. Compound **199** can also increase hyaluronic acid synthases in fibroblasts [55] and showed antibacterial activity inhibitory to *S. aureus* [26].

## 7. Non-Ribosomal Peptides and Bioactivities

Astins constitute a specialized class of chlorinated cyclic pentapeptides featuring both proteinogenic and non-proteinogenic amino acid residues. Their structural diversity predominantly stems from site-specific chlorination patterns on conserved proline moieties. Three astin congeners have been characterized in *Ostropomycetidae* lichens, including astin C (**200**), astin F (**201**), and astin G (**202**), all isolated from *Cyanodermella asteris* L. Jahn & Ludwig-Müll. (Figure 8e) [169,170]. Compound **200** exhibited dual pharmacological properties of anti-inflammatory activity [170], the ability to induce apoptosis in activated T cells, and its potential use in the treatment of colonic inflammation [171]. Both compounds (**200**, **202**) could enhance plant biomass accumulation when individually supplemented in growth media [169].

## 8. Conclusions and Perspectives

Lichens, representing Earth’s predominant symbiotic flora, exhibit remarkable biological adaptability, physiological diversity, and chemical distinctiveness. These extraordinary organisms constitute a vital reservoir of novel bioactive compounds with multifaceted potential. The advanced techniques over recent decades have tremendously pushed the breakthroughs in pharmacological investigations of lichenized species. Their diverse secondary metabolites have established lichens as indispensable natural resources for pharmaceutical development, nutraceutical applications, and industrial biotechnology [77]. Contemporary reviews on lichen-derived drug discovery and lichen biology have emphasized cutting-edge innovations and breakthroughs in symbiotic biology and biosynthetic pathway elucidation [172]. One artificial key to the lichen genera from China have promoted research of lichen resources in China [173]. Of particular interest, the *Ostropomycetidae* subclass, the lichenized fungi Lecanoromycetes’ second-largest taxonomic division, serves as a prolific source of structurally unique compounds with broad bioactivity profiles. Despite a systematic investigation of merely 41% of its taxonomic families across nine orders, the exceptional chemodiversity and demonstrated biological efficacy within this subclass underscore its substantial research potential (Figure 2, Table 1). In recent years, a number of new species of this subclass continue to be discovered, such as from *Gomphillaceae* [174], *Graphidaceae* [175,176,177,178], *Megasporaceae* [179,180], and *Pertusariaceae* [94,181]. However, current exploration limitations primarily stem from the technical challenges in axenic cultivation of symbiotic strains and slow growth, low biomass availability, and factors that have historically deterred the comprehensive phytochemical investigation of lichens.

Rapid development of biotechnological tools is providing a novel opportunity to exploit the compounds from *Ostropomycetidae* lichens for industrial utilization. Culturing the symbionts and the molecular genetics modulation of lichen gene regulation are recognized to enhance the production of target metabolites. The convergence of multi-omics technologies with synthetic biology facilitates the heterologous expression of lichen-derived biosynthetic gene clusters in the tractable fungal hosts. This paradigm shift addresses critical challenges in sustainable metabolite production by decoupling compound biosynthesis from traditional lichen cultivation constraints [182,183]. Future research directions should prioritize (1) promoting biodiversity expansion via novel species discovery to enhance structural novelty potential; (2) applying advanced cultural strategies for targeted metabolite production through controlled symbiont cultivation; (3) achieving genome sequences to facilitate biosynthetic gene cluster identification and functional annotation for more gene resources; (4) applying synthetic biology to employ fungal chassis systems for heterologous compound biosynthesis; and (5) high-throughput screening platforms for activity-guided isolation of therapeutic candidates. The convergence of metabolic engineering and synthetic biology presents unprecedented opportunities to overcome traditional production bottlenecks. Concurrent advancements in multi-omics technologies and CRISPR-mediated pathway engineering are poised to revolutionize our understanding of lichen-derived biosynthetic pathways, enabling precise manipulation of chemical, physiological, and biotechnological processes in these complex symbiotic systems.

## Figures and Tables

**Table 1:** Outline of bioactivities of compounds and their producers in *Ostropomycetidae*.

Bioactivity	Compounds	Lichen Species	References
Antitumor	Haematommic acid (**4**)	*A. pakistanica*	[19]
	Olivetoric acid (**30**)	*Ochrolechia* spp.	[39,41]
	Baeomycesic acid (**38**)	*Siphula* spp.	[48]
	Thamnoliadepside A (**42**)	*T. vermicularis*	[18]
	Atranorin (**55**)	*Siphula* spp., *Baeomyces* spp.	[58]
	Sekikaic acid (**57**)	*S*. *ceratites*, *L. alphoplaca*	[61]
	Alectorialic acid (**62**)	*A*. *pakistanica*	[19]
	Gyrophoric acid (**63**)	*P. mccroryae*, *O. androgyna*, *D. diacapsis*, *A. pakistanica*	[69]
	α-alectoronic acid (**75**)	*O. parella*, *A. radiosa*	[35]
	Physodic acid (**76**)	*D. diacapsis*	[84,85]
	Variolaric acid (**77**)	*O. parella*, *A. radiosa*	[35]
	Protocetraric acid (**79**)	*O. androgyna*	[89]
	Stictic acid (**81**)	*Baeomyces* spp., *Pertusaria* spp.	[96]
	Salazinic acid (**88**)	*P. pseudocorallina*	[89]
	(+)-protolichesterinic acid (**109**)	*Ochrolechia* spp.	[110,111]
	5,7-dihydroxy-6-methylphthalide (**114**)	*A. pakistanica*	[19]
	Usnic acid (**115**)	*Baeomyces* spp., *Aspicilia* spp.	[19]
	6-(4,5-dihydroxy-10-methyl-6-oxo-7-undecenyl)-resorcylic acid lactone (**129**)	*B. placophyllus*	[126]
	Graphislactone A (**140**)	*G. prunicola*, *G. cognata*, *G. scripta*	[133]
	Norlichexanthone (**165**)	*P. laeviganda*	[141]
	Pruinosone (**175**)	*D. pruinosum*	[149]
	Diorygmones A–B (**177**–**178**)	*Diorygma* sp.	[151]
	Dasyscyphin F (**185**)	*Stictidaceae*	[153]
	Petasol (**186**) and sporogen AO-1 (**189**)	*S. tricosa*	[159]
	Graphilane (**194**)	*Graphis* sp.	[158]
	Ergosterol peroxide (**196**)	*O*. *parella*, *S. tricosa*	[164]
	Stigmasterol (**197**)	*Aspicilia* sp.	[167]
	Astin C (**200**)	*C. asteris*	[171]
Anti-inflammatory	Barbatinic acid (**47**)	*T. vermicularis*	[47]
	Atranorin (**55**)	*Siphula* spp., *Aspicilia* spp., *Baeomyces* spp.	[59]
	Physodic acid (**76**)	*D. diacapsis*	[82]
	Wedelolactone (**127**)	*O*. *frigida*	[125]
	Ergosterol peroxide (**196**)	*O*. *parella*, *S. tricosa*	[163]
	Stigmasterol (**197**)	*Aspicilia* sp.	[167]
	Astin C (**200**)	*C. asteris*	[170]
Antibacterial	Squamatic acid (**37**)	*Baeomyces* spp., *Thamnolia* spp.	[20]
	Baeomycesic acid (**38**)	*Siphula* spp.	[117]
	Gyrophoric acid (**63**)	*Ochrolechia* spp., *P. mccroryae*,	[67]
	Protocetraric acid (**79**)	*O. androgyna*	[36,87]
	Lobaric acid (**73**)	*T. vermicularis*, *L*. *alphoplaca*	[80]
	Fumarprotocetraric acid (**80**)	*O. androgyna*	[36]
	Stictic acid (**81**)	*Baeomyces* spp., *Pertusaria* spp., *Diorygma* sp.	[36]
	Hexadecanoic acid (**100**)	*P. caesiopruinosa*	[106]
	Usnic acid (**115**)	*Baeomyces* spp., *Aspicilia* spp.	[16,19]
	Didymic acid (**116**)	*P. flavens*	[120]
	Wedelolactone (**127**)	*O*. *frigida*	[124]
	6,8-dihydroxy-3-hydroxymethylisocoumarin (**135**)	*G*. *proserpens*	[129]
	Vinetorin (**168**)	*Pertusaria* sp.	[142]
	Elsinochrome A (**174**)	*G. elongata*	[146]
	Diorygmone B (**178**)	*Diorygma* sp.	[26]
	Diorygmones C–D (**180**–**181**)	*D. pruinosum*	[26]
	Dasyscyphin F (**185**)	*Stictidaceae*	[155]
	Campesterol (**198**)	*Aspicilia* sp.	[168]
	*β*-sitosterol (**199**)	*T. vermicularis*, *D. pruinosum*	[168]
Antifungal	Methyl-3-chloro-2-hydroxy-4-methoxy-6-pentylbenzoate (**16**)	*P. dactylina*	[27]
	Lecanoric acid (**29**)	*Siphula* spp., *Diploschistes* spp.	[36]
	Divaricatic acid (**31**)	*Pertusaria* spp.	[43]
	Gyrophoric acid (**63**)	*Ochrolechia* spp., *P. mccroryae*, *D. diacapsis*, *A. pakistanica*	[67]
	Xylarinic acid A (**103**)	*G. handelii*	[107]
	Pruinosone (**175**) and hydroxypruinosone (**176**)	*D. pruinosum*	[149]
Antiviral	Sekikaic acid (**57**)	*S*. *ceratites*, *L. alphoplaca*	[64]
	Handelone (**59**)	*G. handelii*	[66]
	Variolaric acid (**77**)	*O. parella*, *A. radiosa*	[35]
	Usnic acid (**115**)	*Baeomyces* spp., *Aspicilia* spp.	[119]
	6,8-dihydroxy-3-hydroxymethylisocoumarin (**135**)	*G*. *proserpens*	[129]
Antioxidant	Orsellinic acid (**6**)	*Diploschistes* spp., *O. frigida*	[24]
	Prephenic acid (**8**), hypoxyphenone (**9**), and tetrafucol A (**28**)	*O. frigida*	[24]
	Lecanoric acid (**29**)	*Siphula* spp., *Diploschistes* spp.	[24,38]
	Cyperine (**36**)	*P. contortuplicata*	[24]
	Atranorin (**55**)	*Siphula* spp., *Baeomyces* spp.	[38]
	Aekikaic acid (**57**)	*S*. *ceratites*, *L. alphoplaca*	[62]
	Gyrophoric acid (**63**)	*Ochrolechia* spp., *P. mccroryae*, *D. diacapsis*, *A. pakistanica*	[67]
	Lobaric acid (**73**)	*T. vermicularis*, *L*. *alphoplaca*	[80]
	Fumarprotocetraric acid (**80**)	*O. androgyna*	[91,92]
	Norstictic acid (**82**)	*Siphula* spp., *Aspicilia* spp., *Graphis* spp.	[79]
	3,6,9,12-tetraoxapentacosanoic acid (**101**), 18-hydroxylinoleic acid (**102**), wedelolactone (**127**), and Diospyrol (**173**)	*O. frigida*	[24]
	Stigmasterol (**197**)	*Aspicilia* sp.	[167]
Anti-angiogenic	Olivetoric acid (**30**)	*Ochrolechia* spp.	[40]
Anti-neurodegenerative diseases	Methylbenzoic acids (**10**,**11**,**12**) and vermicularin (**41**)	*T*. *vermicularis*	[25]
	Baeomycesic acid (**38**)	*Siphula* spp.	[25]
Antitubercular	Acremonidin E (**25**)	*Graphis* sp.	[29]
	Protocetraric acid (**79**)	*O. androgyna*	[88]
Anti-herbivore	Protocetraric acid (**79**)	*O. androgyna*	[57]
	Stictic acid (**81**)	*Baeomyces* spp., *Pertusaria* spp., *Diorygma* sp.	[57]
	Norstictic acid (**82**)	*Siphula* spp., *Aspicilia* spp., *Graphis* spp.	[57]
	(+)-aspicilin (**128**)	*Aspicilia* spp.	[57]
Antitrypanosomal	Lichesterinic acid (**107**) and (+)- protolichesterinic acid (**109**)	*Ochrolechia* spp.	[109]
	Dasyscyphin C (**183**)	*Stictidaceae*	[155]

## Data Availability

The data presented in this study are available in this manuscript, and constructs can be requested from the corresponding author.
